# Simple discovery of bacterial biocatalysts from environmental samples through functional metaproteomics

**DOI:** 10.1186/s40168-017-0247-9

**Published:** 2017-03-03

**Authors:** Premankur Sukul, Sina Schäkermann, Julia E. Bandow, Anna Kusnezowa, Minou Nowrousian, Lars I. Leichert

**Affiliations:** 1 Ruhr-Universität Bochum, Institute for Biochemistry and Pathobiochemistry – Microbial Biochemistry, Universitätsstr. 150, 44780 Bochum, Germany; 2 Ruhr-Universität Bochum, Applied Microbiology, Universitätsstr. 150, 44780 Bochum, Germany; 3 Ruhr-Universität Bochum, Lehrstuhl für Allgemeine und Molekulare Botanik, Universitätsstr. 150, 44780 Bochum, Germany

**Keywords:** Zymogram, Lipase, Biocatalyst, Metagenomics, Metaproteomics

## Abstract

Bacterial biocatalysts play a key role in our transition to a bio-based, post-petroleum economy. However, the discovery of new biocatalysts is currently limited by our ability to analyze genomic information and our capacity of functionally screening for desired activities. Here, we present a simple workflow that combines functional metaproteomics and metagenomics, which facilitates the unmediated and direct discovery of biocatalysts in environmental samples. To identify the entirety of lipolytic biocatalysts in a soil sample contaminated with used cooking oil, we detected all proteins active against a fluorogenic substrate in sample’s metaproteome using a 2D-gel zymogram. Enzymes’ primary structures were then deduced by tryptic in-gel digest and mass spectrometry of the active protein spots, searching against a metagenome database created from the same contaminated soil sample. We then expressed one of the novel biocatalysts heterologously in *Escherichia coli* and obtained proof of lipolytic activity.

**Electronic supplementary material:**

The online version of this article (doi:10.1186/s40168-017-0247-9) contains supplementary material, which is available to authorized users.

## Main text

A conceptually straightforward way to identify new microbial biocatalysts is the screening of a multitude of organisms isolated from an environmental sample for a desired enzymatic activity [1]. However, due to our inability to cultivate the vast majority of microorganisms in the lab, such a screening will miss potentially more than 99% of organisms present in a given environmental sample [2]. To counter this problem, DNA-based, culture-independent approaches have now become the state-of-the-art in biocatalyst discovery. These methods rely on library-based screening efforts, where an expression library from environmental DNA is screened for a certain activity. Coining the term metagenome, this concept was introduced by Handelsmann and co-workers [3] and has been used e.g., in large-scale projects to identify lipolytic enzymes from soil metagenomes [4]. This approach typically involves screening hundreds of thousands of clones, and the number of biocatalytically active proteins discovered is dependent on the library size. An alternative is the in silico search for homologs of known biocatalysts in metagenomic datasets, a method we have recently employed ourselves [5], and which is comprehensively reviewed in [6]. This method uses known structural motifs to find novel enzymes in sequence databases. Rapid advances in sequence-based metagenomics and a plethora of publicly available DNA data have led to a widespread adoption in the scientific community. However, it can be argued that in silico screening loses the immediacy of an activity-based, i.e., structurally unbiased, discovery by adding an additional layer of abstraction in the form of DNA-sequence data.

Here, we present a functional metaproteomic approach as a method for rapid enzyme discovery. This method combines the immediacy of an activity-based screening with the independence from lab-cultivability of “meta-omic” approaches. This approach is conceptually comprehensive as it has the potential to discover all enzymes that exhibit an activity that can be screened for in an environmental sample, in principle facilitating the discovery of novel structure-function pairs. The method does not rely on a comprehensive evaluation of the metagenome and metaproteome data but rather utilizes both to simplify the discovery of proteins exhibiting a desired enzyme activity.

Metaproteomics is quickly becoming a well-established high-throughput “meta-omic” approach to study microbial ecology, as recently reviewed in [7] and [8]. Metaproteomics was developed by Bond and Wilmes to mine microbiomes for novel proteins from previously uncultured organisms [9], and one of its earliest applications was the functional study of biocatalysts that degrade organochloride pollutants [10].

**Fig. 1:**
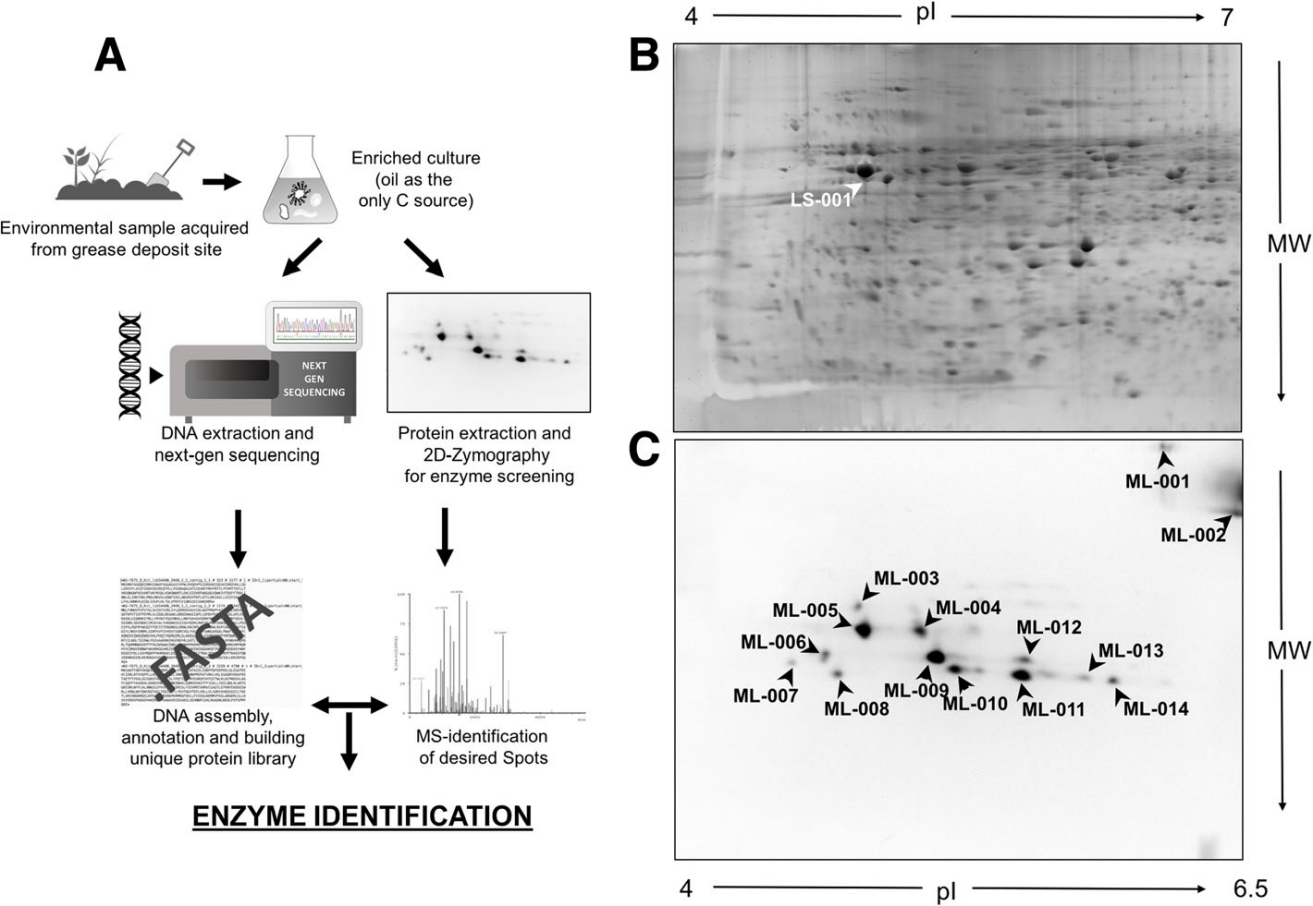
Functional metaproteomics as a tool to discover biocatalysts. **a** Schematic representation of the functional metaproteomics workflow. Metagenomics and functional metaproteomics combine the immediacy of an activity-based approach, while still retaining the comprehensive information of the metagenome. Optimization of DNA and protein extraction protocols can be found in Additional file 1: Figure S2 **b** 2D gel electrophoresis of the enriched sample stained with RuBPS Protein Gel Stain. LS-001 was excised as landmark spot. **c** In-gel activity assay for identifying lipolytic enzymes in the metaproteomic sample. Methylumbelliferyl-butyrate was hydrolyzed by lipolytic enzymes present in the gel. Resulting methylumbelliferone was detected under ultraviolet light. Fourteen spots (ML-001–ML-014) were manually excised for subsequent mass spectrometric analysis. Representative results shown, results of both technical replicates can be found in Additional file 1: Figure S3

We also used a functional metaproteomic approach to identify lipolytic enzymes from environmental sources. Thus, we collected samples from a site where we expected microorganisms harboring these activities to dwell in large numbers. We harvested one oil-contaminated soil sample from a restaurant’s used cooking oil disposal site and used it for enriching microorganisms with lipolytic activity. Proteins and DNA were isolated from the same sample (see Additional file 1 for more details). The proteins (600 μg of protein) were separated by two-dimensional (2D) polyacrylamide gel electrophoresis. After separation, proteins were refolded in the gel and an in-gel activity assay based on the fluorogenic lipase substrate *para*-methylumbelliferyl butyrate (pMUB) was performed. pMUB is a substrate that can be used to detect a wide variety of lipolytic and hydrolytic enzymes with high sensitivity [11]. Lipolytic enzymes present in the gel hydrolyzed pMUB and released butyric acid and p-methylumbelliferone, which is a fluorescent dye that can be detected under ultraviolet light. The intensity of the spot is dependent both on the quantity of the protein and its activity. With this method, we identified 14 lipolytically active spots in our protein sample (Fig. 1). These experiments were performed in duplicates. The fluorescing protein spots were then excised from the gels, tryptically digested and analyzed by mass spectrometry. Mass spectrometry-based protein identification is facilitated by searchable databases of predicted masses that arise from the fragmentation of tryptic peptides. We therefore created such a database from the metagenomics sequences we obtained from the DNA isolated from the sample. Through next-generation sequencing, we obtained a high quality DNA dataset with most sequences showing a Phred score higher than 35 indicating a base call accuracy close to 99.99% [12]. The sequenced raw data was assembled to recover original genome information and to predict proteins using the assembly software SPAdes v3.1.1 [13] and annotated using PROKKA v1.10 [14]. Prokka uses Prodigal to identify coding sequences in the assembled metagenome [15] and then transfers the annotation of the most significant match from a hierarchy of data sources to these sequences. Coding sequences that do not match are labeled as hypothetical protein. The obtained database contained approximately 161,000 proteins, of which 37.6% were annotated as hypothetical proteins with unknown function.

**Fig. 2:**
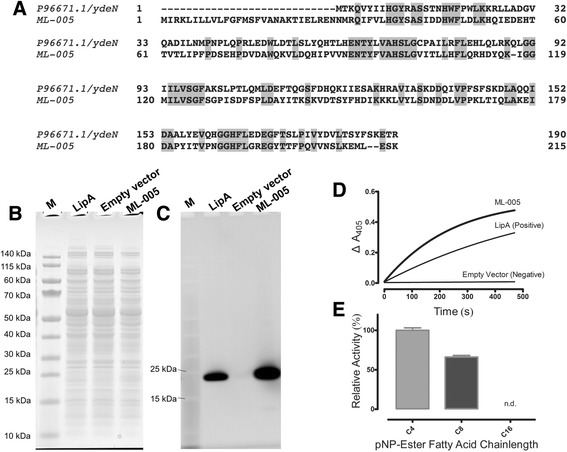
ML-005 is a novel esterase. **a** ML-005 is a distant relative (35% identity) of the uncharacterized putative hydrolase YdeN of *B. subtilis*. **b** Heterologous protein expression of ML-005 and lipase A LipA from *B. subtilis* (positive control) in *E. coli* from a plasmid was induced with 1 mM IPTG. *E. coli* carrying the empty vector served as negative control. Cells were disrupted by sonication and crude extracts were subjected to SDS PAGE, the protein content visualized by coomassie staining. **c** Lipid hydrolyzing activity was detected through in-gel zymography in the same crude cell lysates. The in-gel activity assay shows substrate conversion for positive control LipA from *B. subtilis* (23 kDa) and ML-005 (24.5 kDa) while a negative control of an extract of *E. coli* carrying the empty vector shows no activity. **d** Crude extract of *E. coli* expressing ML-005 hydrolyzes *para*-nitrophenyl-butyrate. Crude extract of *E. coli* expressing LipA from *B. subtilis* served as positive control, crude extract of *E. coli* containing the empty vector as negative control. Representative results are shown, results of all biological replicates can be found in Additional file 1: Figure S4. **d** Substrate specificity of purified ML-005 indicates a preference towards short-chain (C_4_) and medium-chain length (C_8_) *para*-nitrophenyl esters typical for esterases, no activity towards long-chain (C_16_) esters could be detected (n.d.). Specific activity of ML-005 towards *para*-nitrophenyl butyrate was 14.1 U mg^−1^

This customized database was then used to analyze the mass spectra obtained from the 14 lipolytically active protein spots (Additional file 1: Table S1). Among these protein spots, we identified 2 serine-hydrolases, homologous to known lysophospholipase TesA from *Pseudomonas* species (ML-009 and ML-010). Additionally, 6 uncharacterized proteins, all homologous to a thioesterase from *Pseudomonas* species were found in the gel (ML-002, ML-003, ML-007, ML-008, ML-010, and ML-014). In total, 9 distinct primary structures of thioesterases, which matched the mass spectra generated from these spots, were present in our annotated metagenome database. These were all highly similar with minute differences (Additional file 1: Figure S1) and could not be unequivocally matched to the protein digests. All of these proteins are members of the family of SGNH hydrolases (named for their conserved and characteristic serine, glycine, asparagine, and histidine residues [16]), which are known to show lipolytic activity towards ester substrates [17]. Especially, TesA from *Pseudomonas* is known to have a preference for ester substrates with short- and mid-range carbon chain length [18]. In addition, we identified hydrolase ML-005, which is distantly homologous (35% identity) to the as of yet uncharacterized putative hydrolase YdeN (UniProtKB = P96671.1) from *Bacillus subtilis*. Bioinformatic analysis of ML-005 revealed a putative conserved alpha/beta hydrolase domain in this protein (Accession No. COG3545 at NCBI [19]) (Fig. 2a).

To verify the biocatalytic activity of the uncharacterized hydrolase ML-005, its DNA sequence was synthetized based on the metagenome data and cloned into *Escherichia coli* in an IPTG-inducible pBR322 -based expression vector with a *tac* promotor. The protein was then heterologously expressed in *E. coli* and its lipolytic activity confirmed through in-gel zymography (Fig. 2b, c). Furthermore, crude extract of *E. coli* expressing ML-005 showed high activity in a standard lipase/esterase enzyme assay, using p-nitrophenyl butyrate as a substrate (Fig. 2d). Lipid hydrolyzing enzymes can be categorized as lipase or esterases, with esterases typically preferring short-chain and lipases preferring long-chain fatty acid esters as substrates. We thus cloned the gene encoding ML-005 into a pET-based expression vector containing a T7-promotor, fusing a C-terminal His_6_-tag to the protein. We then expressed ML-005 in *E. coli* BL21 and purified it to homogeneity to test its reactivity towards *para*-nitrophenyl esters with fatty acids of differing chain-lengths. While ML-005 was effective in hydrolyzing short-chain (C_4_) and medium-chain length (C_8_) esters, we were not able detect any activity towards the long-chain p-nitrophenyl palmitate (C_16_), indicating that ML-005 is an esterase (Fig. 2e).

In conclusion, functional metaproteomics is an efficient tool to directly discover biocatalytic activity in the proteome of an environmental sample. The limitations of our approach pertain to the difficulties inherent in the isolation of proteins and DNA from environmental samples [20–24]. The complete phylogenetic diversity of a sample could only be harnessed if all DNA and all proteins expressed in the sample would be isolated. The method furthermore depends on an effective in-gel refolding of the biocatalyst and the availability of zymographic assays [25] that can be adapted to screen environmental samples for a certain biocatalytic activity.

Our results show that a simple workflow that combines 2D gel-based proteomics, functional screening, and metagenome-based protein identification makes it possible to identify novel lipolytic enzymes, an important class of biocatalysts, on the protein level, harnessing the phylogenetic diversity found in an environmental sample from a used cooking oil disposal site. We validated our approach by the heterologous expression and purification of the newly discovered and previously unknown esterase ML-005.

## Additional file

Additional file 1: Supplementary methods [27–29], figures [30] and table [26]. (PDF 2013 kb)

## Abbreviations

2D: Two dimensional
pMUB: *para*-methylumbelliferyl butyrate
