# Supplementary material for: Simple discovery of bacterial biocatalysts from environmental samples through functional metaproteomics
Source: Microbiome. 2017 Mar 3;5:28. doi: 10.1186/s40168-017-0247-9 (PMC5335836; doi:10.1186/s40168-017-0247-9)
Supplement: Additional file 1: — Supplementary methods [27–29], figures [30] and table [26]. (PDF 2013 kb) [file 40168_2017_247_MOESM1_ESM.pdf]

Supplementary Information

Materials and Methods

Environmental sample preparation

Soil samples were collected from a used cooking oil deposit site of a restaurant (Dortmund, Germany, Latitude/Longitude: 51.528874,7.458762) with the assumption that long-term exposure to oil contamination led to a natural selection for microbes capable of metabolizing oil. Each soil sample was homogenized by grinding and sieving (2 mm particle size). Lipase-producing bacteria were enriched by inoculating 1 L of modified Belitzky Minimal Medium [27] with 1 g of soil. The medium was supplemented with olive oil (2 %) as the sole carbon source. Upon growth (3 days), the cultures were centrifuged to acquire the pellet and stored at -80 °C.

Protein Extraction and 2D Gel Electrophoresis

The cell pellets of oil-metabolizing organisms were disrupted to acquire the proteome for 2D polyacrylamide gel electrophoresis analysis by a bead-beating method in the presence of a non-ionic detergent, n-Dodecyl β-D-maltoside. Approximately 3-5 ml cell pellet was resuspended in 350 µl Sample Buffer [28] (50 mM Bis-Tris, 50 mM NaCl, 1 mM EDTA, pH 7) and 100 µl of 10 % (w/v) n-dodecyl β-D-maltoside. Glass beads (diameter 0.5 mm) in a volume approximating the size of the cell pellet were added. The suspension was vortexed 5 min and incubated for 20 min on ice. Subsequently, it was centrifuged at 16,000 x g at 4° C. Proteins were precipitated from the supernatant using a methanol/chloroform extraction. For this purpose, 3 volumes of water, 4 volumes of methanol and 1 volume of chloroform were added to 1 volume of protein-containing supernatant. The mixture was vortexed vigorously and centrifuged for 20 min at 16,000 x g and 4° C. The protein precipitate was found at the interface between the two phases. The top phase was removed carefully without disturbing the precipitate. Then 400 µl of methanol

was added and the sample vortexed vigourously before centrifuging the sample in the above-mentioned conditions. The protein precipitate, now at the bottom of the tube, was washed three times with chilled acetone, before briefly drying the pellet and re-suspending it in DTT-free rehydration buffer (7 M urea, 2 M thiourea, 65 mM CHAPS, 0.5 % v/v Triton X-100, 1.04 % v/v Pharmalyte pH 3-10 and traces of bromophenol Blue). The protein solution was then separated in a 2 D gel electrophoresis using a protocol previously published [29] with the following modifications: the equilibrating solution used to prepare the isoelectric focussing IPG gel strip neither contained DTT nor iodoacetamide. 2D gels not intended for zymography were stained with RuBPS Protein Gel Stain and proteins were visualized on a Typhoon laser scanner (GE Healthcare, Freiburg, Germany). 2D gels were performed in duplicates.

Zymography to detect lipolytic enzymes

After protein separation, the zymography 2 D gels were incubated for 3 × 10 min in 400 ml Triton X-100 (2.5 % v/v) to remove interfering SDS from the gels. The gel was then incubated in 400 ml phosphate buffer (50 mM, pH 7) followed by incubation of the gel in 400 ml 100 µM *p*-methylumbelliferyl butyrate (pMUB) solution in phosphate buffer. pMUB is a fluorogenic substrate for lipolytic enzymes. A lipase or an esterase hydrolyses the substrate to butyric acid and the fluorescent *p*-methylumbelliferone, which can be detected under UV light. Fluorescing spots were manually excised for subsequent mass spectrometry on a UV table.

Mass Spectrometry

The excised protein spots were treated twice for 15 min with 400 µl 20 mM ammonium bicarbonate in 30 % acetonitrile. Subsequently protein spots were reduced in 50 µl 10 mM DTT at 60 °C for 45 min and then alkylated with 50 mM iodoacetamide at 25 °C in the dark. The gel spots were then washed twice for 5 min with 400 µl 20 mM ammonium bicarbonate in 30 %

acetonitrile and dried completely in a vacuum centrifuge. The spots were tryptically digested overnight at 37 °C using 6.25 ng/µl trypsin in 20 mM ammonium bicarbonate, 30 % acetonitrile. Trypsin was obtained from Promega, Fitchburg, USA. Peptides were eluted into ultrapure water containing 0.1 % trifluoroacetic acid using an ultrasonic bath for 15 min. Eluates were loaded on a trap column (C_{18}, pore size 100 Å, particle diameter 5 µm, inner diameter 180 µm, length 20 mm, Waters, Eschborn, Germany) and were then eluted using a gradient of acetonitrile with 0.1 % formic acid (flow rate 350 nl/min, linear gradient 0.5-5 % in 4 min, 5-60 % in 45 min) from an analytical column at 40 °C (C_{18}, pore size 130 Å, particle diameter 1.7 µm, inner diameter 75 µm, length 150 mm) followed by mass spectrometry using a Synapt G2-S instrument (Waters). Spectra were recorded in positive resolution mode over a mass range of 50-1800 m/z with 1 s/scan using the MS^E technology with a collision ramp of 14-35 eV. The parameters used for the NanoLockSpray source were as follows: Capillary voltage of 2.2 kV; Sampling cone voltage of 30 V; source temperature of 100 °C; desolvation temperature of 150 °C; cone gas flow of 50 L/h and desolvation gas flow of 500 L/h. Leucine enkephaline, serving as lock mass analyte (m/z=556.2771), was fed through the lock spray channel.

Data were analyzed using the ProteinLynxGlobalServer 2.5.2 (PLGS; Waters) software. Mass spectra were processed using the parameters as follows: chromatographic peak width set to automatic; MS ToF resolution set to automatic; lock mass window of 0.25 Da; low energy threshold of 50 counts; elevated energy threshold of 15 counts; elution time of 5-45 min; intensity threshold of 500 counts.

A metagenomics-derived database was used for protein identification using the parameters as follows: peptide tolerance set to automatic; fragment tolerance set to automatic; minimal fragment ion matches per peptide of 2; minimal fragment ion matches per protein of 2; minimal

peptide matches per protein of 1; maximum protein mass of 250 kDa; primary digest reagent was trypsin; missed cleavages was set to 2; carbamidomethyl C was set as fixed modifications; deamidation N and Q, oxidation M were set as variable modifications and false positive rate was set at 4 %.

The mass spectrometry proteomics data along with the metagenomics-derived database, have been deposited to the ProteomeXchange Consortium via the PRIDE partner repository with the dataset identifier PXD005148. Username for review purposes: reviewer00553@ebi.ac.uk Password: WJRenyTK. The deposited data includes the unprocessed MS data, as well as the ProteinLynxGlobalServer output after processing. The output data includes tables in .csv (comma separated values) format for all identified fragment-ions, peptides and proteins, respectively. Furthermore, reports for the data base search (IA_log and IA_workflow) are given.

DNA extraction, sequencing, and annotation

High quality, unsheared DNA was extracted by combining a bead-beating method with a commercial kit-based extraction. The enriched cell-pellet was initially disrupted using glass beads and 450 µl STES buffer (0.1 % SDS, 0.2 M Tris-HCl pH 7.5, 0.01 M EDTA and 0.5 M NaCl). Upon centrifugation (16,000 x g, 10 min, RT), the cell lysate was transferred into QIAamp® DNA Mini and Blood Mini Kit Filter (QIAGEN GmbH, Hilder, Germany). The DNA was then isolated according to the standard manufacturer's protocol. The acquired DNA (OD260/280=1.9 and an OD260/230=2.0) was sequenced at GATC (Constance, Germany) in 300 bp paired end reads using the MiSeq platform.

Reads were trimmed with the program trim_fastq1.pl with a minimum read length after trimming of 150 nt and the minimum quality for cutoff set to a phred score of 20. The sequence data was assembled using SPAdes (v3.1.1) with k-values of 21, 33, 55, 77, 99, and 127 and annotated and

translated into an amino-acid sequence library using Prokka (v1.10). SPAdes was invoked using ./spades.py -k 21,33,55,77,99,127 --careful --only-assembler --pe1-12 paired_end_library.fastq --s1 single_read_library.fastq -o NG7675_spades1. Prokka was invoked using ./prokka --outdir output_directory --prefix NG7675_spades1_prokka --metagenome NG7675_spades1_scaffolds.fasta.

The resulting database was used for identifying lipases from the 2D zymograms.

Sequencing data of the sample was submitted to the European Nucleotide Archive (www.ebi.ac.uk/ena) under project number PRJEB16064 and sample accession numbers ERP017906.

Heterologuos expression of metaproteomic lipase ML-005 and enzyme activity assays

The protein sequence for lipase ML-005 was re-translated to a codon-optimized gene sequence for expression in *E. coli* and synthesized (GeneArt, Regensburg, Germany). The gene was then cloned into a pTAC-MAT-Tag-2 (Sigma Aldrich, St. Louis, MO, USA) based expression vector[4] and in the pET22b expression vector with a T7 promoter system and a C-terminal His_6-tag for purification. The vectors were transformed into *Escherichia coli* XL1 Blue (pTAC-MAT-Tag-2) and BL21 (pET22b) using heat shock transformation. For crude cell extract generation, the XL1 Blue cells containing ML-005, *Bacillus subtilis* LipA, or an empty vector were grown overnight at 37 °C in LB liquid medium with 100 µg/ml ampicillin for selection. Next day, a secondary culture (10 ml LB supplemented with ampicillin) was inoculated with the overnight culture to an OD_{600} of 0.05. IPTG (1 mM) was added at OD_{600} of approximately 0.5 and the culture incubated for another 2-3 h. The cultures were centrifuged (13,000 x g, 2 min, 4° C) and the acquired cell pellet was resuspended in Tris-HCl buffer (pH 7). The cell suspension was disrupted through sonication (amplitude: 80 %, Cycle: 0.5 s, 3 x 1 min) using a Vial Tweeter instrument (Hielscher,

Teltow, Germany). The resulting crude extract was centrifuged (13,000 x g, 20 min, 4 °C) to remove cell debris. The supernatant was used for spectrophotometric lipolytic activity determination using pNP-butyrate. The diluted supernatant (2 % v/v in 50 mM Tris-HCl pH 7) was kept at 45 °C. The hydrolysis reaction was initialised by adding 200 µM pNP-ester from a 4 mM stock in aqueous solution with 4% ethanol, 1% acetonitrile, and 0.4% Triton X-100. The enzymatic reaction was measured by monitoring the absorbance at 420 nm in a V-650 spectrophotometer (Jasco, Tokyo, Japan). These experiments were performed with three biological replicates (Figure S4). For purification of ML-005, *E. coli* BL-21 was inoculated in LB medium, supplemented with 100 mg/l ampicillin from an overnight culture at an OD600 of 0.05. At an OD600 of 0.5, overexpression was induced by 1 mM of IPTG and the cell suspension incubated overnight at 20°C under constant shaking. Cells were harvested by centrifugation (45 min at 6,500x g and 4°C.) The resulting pellet was resuspended in approximately 50 ml of Tris-HCl-buffer (50mM, pH 7.1). Cell disruption was carried using the Constant Cell Disruption System (Constant Systems, Low March, UK) at 1.9 kbar three times. The cell lysate was centrifuged for 60 min at 6,500 g and 4°C to get rid of cell debris. The supernatant containing the soluble protein was filtered using 250 ml Filtropur V50 vacuum filters (Sarstedt, Nümbrecht, Germany). An ÄKTApurifier FPLC system (GE Healthcare, Uppsala, Sweden) was used to purify ML-005. A 5 ml HisTrap HP Ni-NTA-column (GE Healthcare, Uppsala, Sweden) was first washed with distilled water and then equilibrated using Buffer A (50 mM sodium phosphate, 300 mM NaCl, pH 8.0). The filtrate was loaded into the column and ML-005 was eluted using 10% and 12% of Buffer B (50 mM sodium phosphate, 300 mM NaCl, 500 mM imidazole, pH 8.0). The resulting protein solution was dialyzed overnight at 4°C against 50 mM sodium phosphate buffer (pH 8) using a a Spektra/Por Dialysis Membrane 12–14 kDa

(Spectrumlabs, Rancho Dominguez, USA). Substrate specificity against *para*-nitrophenyl (pNP) esters was performed as described above, the hydrolysis reaction was initialised by adding 50 µM of the corresponding pNP ester to a 38.3 nM protein solution in 50 mM sodium phosphate buffer (pH 8). The enzymatic reaction was measured over approximately 8 min by monitoring the absorbance at 405 nm in a V-650 spectrophotometer (Jasco, Tokyo, Japan). The activity was calculated from the initial slope of the absorbance. The activity against pNP-butyrate (14.1 $U \cdot mg^{-1}$) was set to 100%.

Supplementary Figures

Figure S1

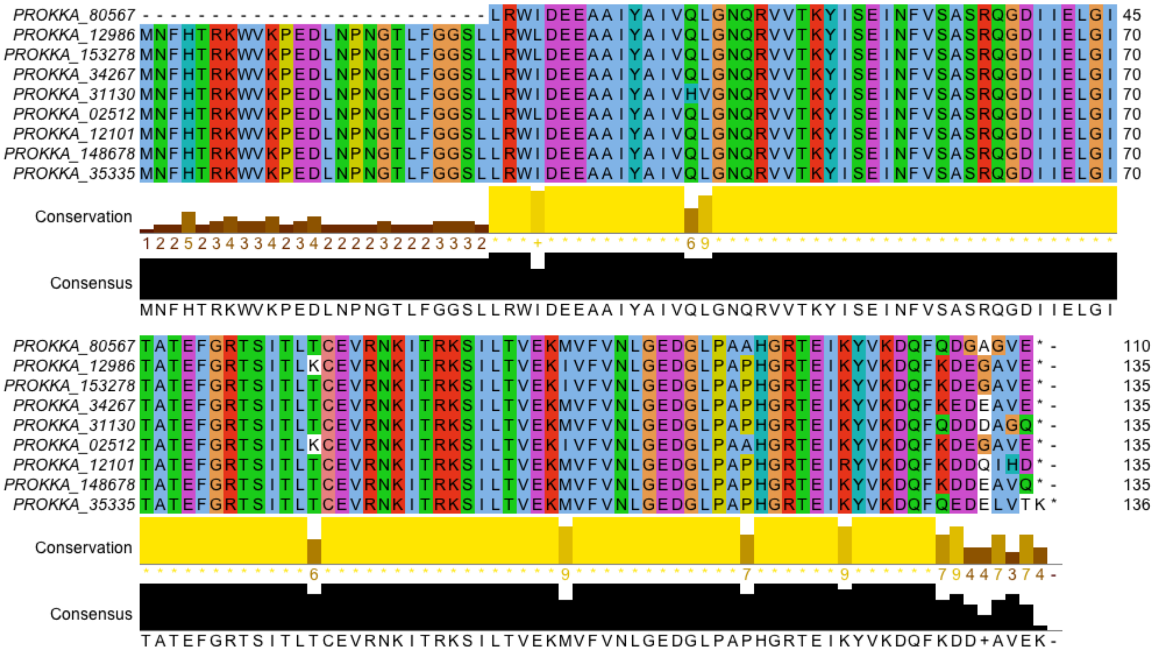

Figure S1: Alignment of nine uncharacterized proteins annotated from the metagenome of our sample. These proteins are homologous to a group of thioesterases from *Pseudomonas* species and MS-based peptide identifications derived from spots ML-002, ML-003, ML-007, ML-008 ML-010 and ML-014 are consistent with this protein group, but cannot discriminate between the individual entries. Alignment visualized using Jalview 2 [30].

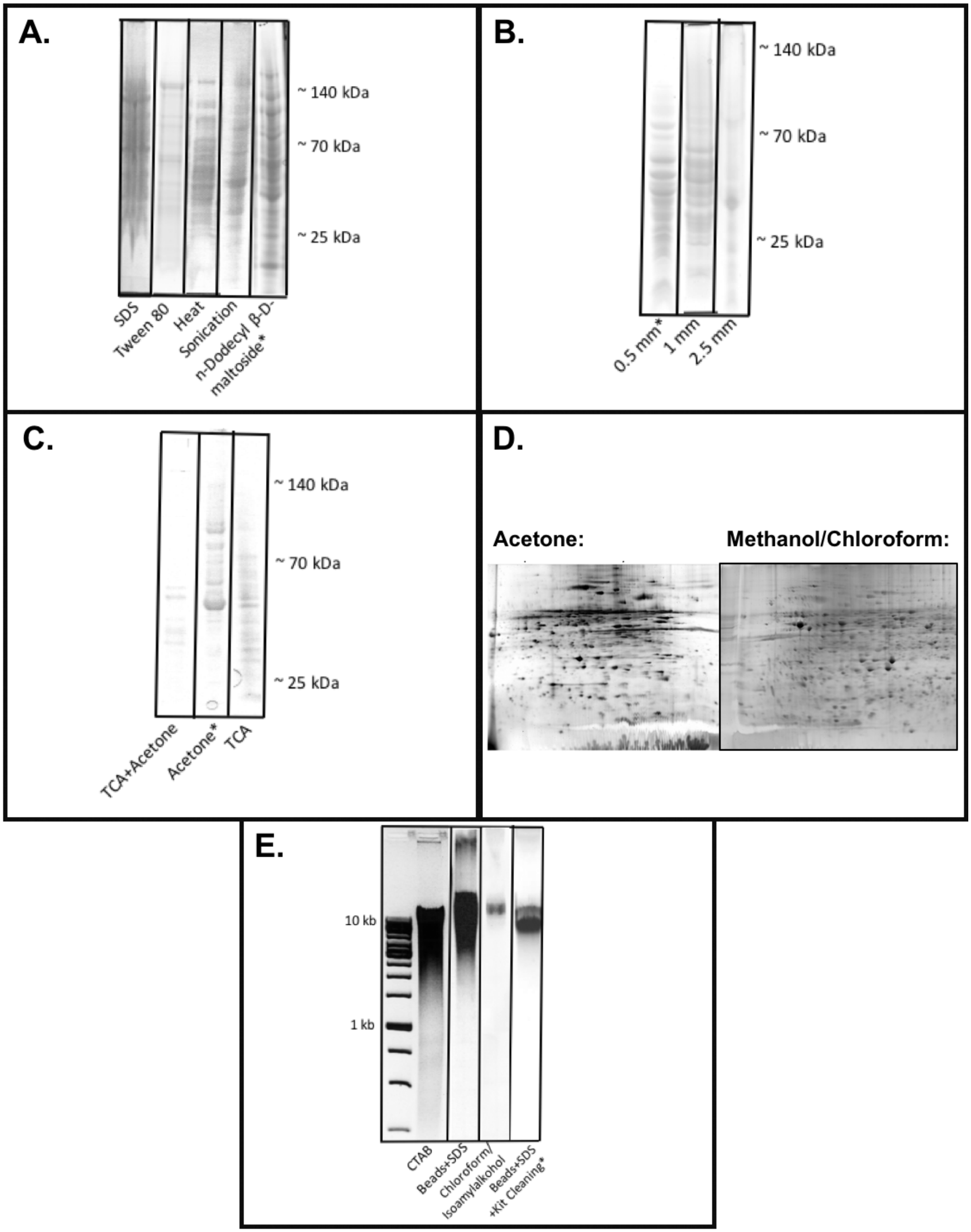
A.
~ 140 kDa
~ 70 kDa
~ 25 kDa
SDS
Tween 80
Heat
Sonication
n-Dodecyl β-D-maltoside*
B.
~ 140 kDa
~ 70 kDa
~ 25 kDa
0.5 mm*
1 mm
2.5 mm
C.
~ 140 kDa
~ 70 kDa
~ 25 kDa
TCA+Acetone
Acetone*
TCA
D.
Acetone:
Methanol/Chloroform:
E.
10 kb
1 kb
CTAB
Beads+SDS
Chloroform/Isoamylalkohol
Beads+SDS +Kit Cleaning*

Figure S2: Optimization of protein and DNA extraction protocols. *denotes that the parameter was deemed as the best for our specific sample and was then implemented in the next optimization step. (**A**) Cell disruption was carried out by physical methods: Sonication (3 x 30 s; amplitude 80; cycle 0.5) and heat (95°C; 5 min); as well as with detergents: SDS (5% v/v, 15 min), tween 80 (1.5% v/v, 40 min incubation) and dodecyl maltoside (see Materials and Methods). Dodecyl maltoside yielded the highest amount of proteins. (**B**) To enhance cell lyses, bead beating was employed. Different diameters of beads were tested to evaluate their efficacy in supporting cell lysis. 0.5 mm glass beads yielded the most protein bands and reduced smearing. (**C**) Protein precipitation methods to remove impurities that lead to band smearing on an SDS PAGE gel were tested. Acetone was initially chosen as the protein precipitation method of choice as it resulted in higher protein recovery than a trichloroacetic acid (TCA) and an acetone + TCA hybrid method. (**D**) However, acetone precipitation resulted in horizontal smearing in 2D PAGE gels. Methanol-chloroform precipitation was finally chosen as alternative to acetone precipitation as it led to higher purity and recovery and better focusing of protein spots. (**E**) To extract unsheared DNA from the environmental sample, we evaluated several DNA extraction methods. In the CTAB method cetrimonium bromide, a surfactant, was used for cell disruption, followed by isopropanol precipitation of DNA. In the chloroform/isoamylalcohol method, DNA was extracted using lysozyme (2mg), SDS (10% v/v) and chloroform/isoamylalkohol (24:1). DNA was then precipitated using ethanol. DNA was also extracted using a bead-beating method. 0.5 mm glass beads were used in the presence of 0.1% SDS and subsequent phenol/chloroform extraction. DNA was then precipitated using ethanol. Only the DNA acquired through bead beating method was unsheared. To increase the quality, DNA was further purified by using the

QIAamp® DNA Mini and Blood Mini Kit. This additional step provided unsheared, pure DNA (OD260/280 = 1.9 and an OD260/230 = 2.0) with sufficient quality for sequencing.

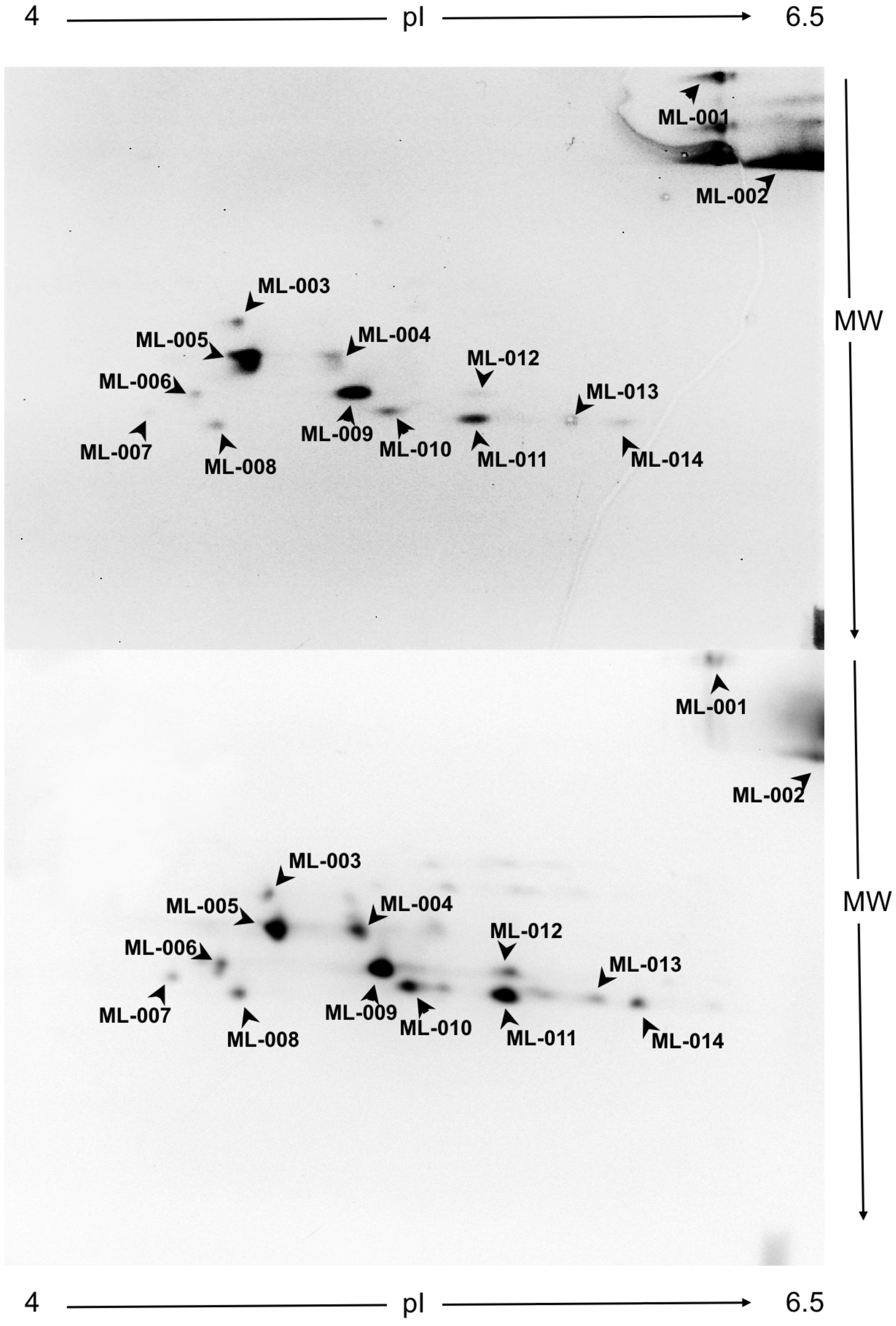
4
pI
6.5
MW
ML-001
ML-002
ML-003
ML-004
ML-005
ML-006
ML-007
ML-008
ML-009
ML-010
ML-011
ML-012
ML-013
ML-014
ML-001
ML-002
ML-003
ML-004
ML-005
ML-006
ML-007
ML-008
ML-009
ML-010
ML-011
ML-012
ML-013
ML-014
MW
4
pI
6.5

Figure S3: Technical replicates of zymograms from 2D gels from the protein extract of our environmental sample. Protein extract was separated and in-gel activity assay was performed in independent experiments on different days.

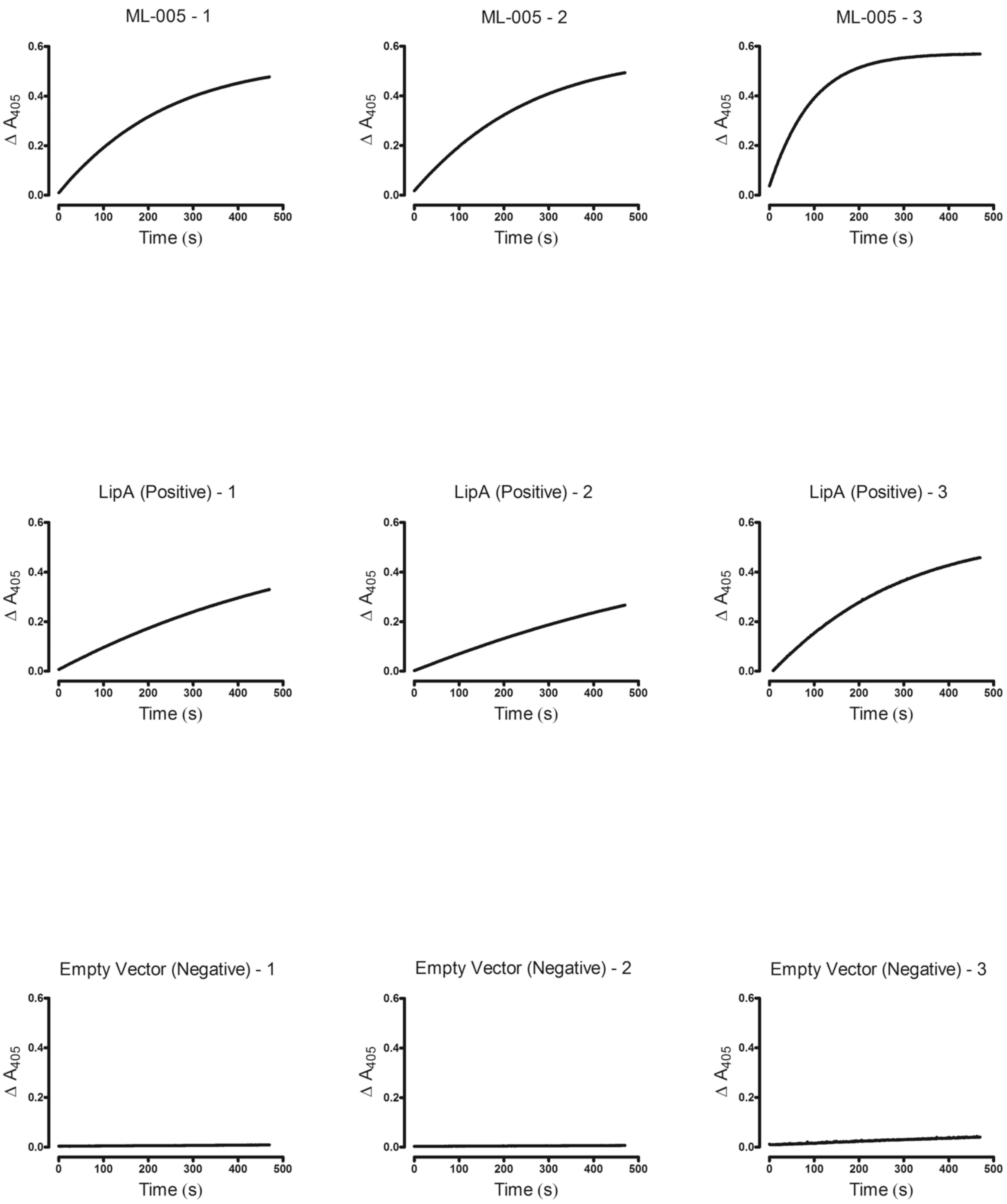

Figure S4: Biological replicates of the *para*-nitrophenyl butyrate hydrolysis assays with crude extract from *E. coli* expressing the novel esterase ML-005, Lipase A (LipA) from *B. subtilis* as positive control, or containing empty vector as negative control.

Table S1:

List of identified proteins from 2D gels according to PLGS software. Multiple proteins were identified most likely due to the presence of multiple proteins at the site of activity-based excision of protein spots. Identified proteins that were considered potential lipolytic enzymes are highlighted in bold. Molecular weight (Da) and pI are predicted. Peptides: number of peptides identified. ML-001 to ML-014 represent the 14 lipolytic spots excised from zymography gels. LS-001 is a landmark spot excised from RuBPS stained gels. Spots were tryptically digested and analyzed via mass spectrometry. The full dataset including peptide sequences and raw data is available via ProteomeXchange with identifier PXD005148 [26].

ID	Database ID	Description	mW (Da)	pI (pH)	Peptides
LS-001	PROKKA_92879	Major outer membrane protein P.IB precursor	39796	4.8	99
ML-001	PROKKA_100300	Chaperone protein DnaK	68374	4.7	24
	PROKKA_92465	Chaperone protein DnaK	68358	4.7	25
	PROKKA_22662	Chaperone protein DnaK	37702	4.6	14
	PROKKA_138643	Chaperone protein DnaK	69042	4.6	20
	PROKKA_72460	Chaperone protein DnaK	24543	4.6	11
	PROKKA_138668	Chaperone protein DnaK	24943	4.6	13
	PROKKA_150754	Chaperone protein DnaK	59912	4.6	20
	PROKKA_143530	Chaperone protein DnaK	68343	4.6	22
	PROKKA_139251	Chaperone protein DnaK	68632	4.7	20
ML-002	PROKKA_41331	A-type flagellin	57137	4.5	17
	PROKKA_70441	Hypothetical protein	13678	6.6	4
	**PROKKA_02512**	**Putative acyl-CoA thioester hydrolase**	15058	7.3	1
	PROKKA_90774	Methionine synthase	23142	4.8	4

Table S1 continued:

ID	Database ID	Description	mW (Da)	pI (pH)	Peptides
ML-003	PROKKA_67835	Superoxide dismutase [Fe]	22892	5.5	13
	PROKKA_00382	Isocitrate dehydrogenase [NADP]	45583	5.2	9
	PROKKA_127235	Isocitrate dehydrogenase [NADP]	45572	5.1	9
	**PROKKA_80567**	**Thioesterase superfamily protein**	12138	5.2	4
	PROKKA_38202	Isocitrate dehydrogenase [NADP]	45493	5.3	11
	PROKKA_161078	Isocitrate dehydrogenase [NADP]	45686	5.5	7
	PROKKA_35975	Nucleoside diphosphate kinase	15438	5.4	8
	PROKKA_21910	Ribosome-recycling factor	20640	5.5	8
	PROKKA_18267	50S ribosomal protein L28^APROKKA_135858 50S ribosomal protein L28	8973	12.1	3
	PROKKA_92879	Major outer membrane protein P.IB precursor	39796	4.8	14
	PROKKA_30155	Alpha-ketoglutarate-dependent taurine dioxygenase	12167	5.3	1

Table S1 continued:

ID	Database ID	Description	mW (Da)	pI (pH)	Peptides
ML-004	PROKKA_118556	Superoxide dismutase [Fe]	21960	5.3	4
	PROKKA_48537	Superoxide dismutase [Fe]	21976	5.7	3
	PROKKA_48865	hypothetical protein	8980	5.8	4
	PROKKA_112895	Phage portal protein	37687	5.5	1
	PROKKA_78659	hypothetical protein	152376	5.4	11
	PROKKA_52769	LPS-assembly lipoprotein LptE	22131	8.5	15
ML-005	PROKKA_119255	Superoxide dismutase [Fe]	21990	5.5	14
	PROKKA_123218	Superoxide dismutase [Fe]	21988	5.5	16
	PROKKA_54206	Superoxide dismutase [Fe]	22031	5.5	10
	PROKKA_136888	Superoxide dismutase [Fe]	21950	5.5	10
	**PROKKA_47441**	**Putative hydrolase YdeN**	24540	5.7	9
	PROKKA_120225	Hypothetical protein	15133	6.6	4
	PROKKA_92879	Major outer membrane protein P.IB precursor	39796	4.8	15
	PROKKA_52696	Hypothetical protein	13173	9.6	2
	PROKKA_102188	Outer membrane protein A precursor	17906	4	3
	PROKKA_120445	Peptidase inhibitor I78 family protein	9730	4.5	1
	PROKKA_61135	Hypothetical protein	9338	9.6	2

Table S1 continued:

ID	Database ID	Description	mW (Da)	pI (pH)	Peptides
ML-006	PROKKA_00170	Outer membrane protein W precursor	24683	5.8	10
	PROKKA_92879	Major outer membrane protein P.IB precursor	39796	4.8	25
	PROKKA_126912	Outer membrane efflux protein	10568	9.8	3
	PROKKA_67835	Superoxide dismutase [Fe]	22892	5.5	6
ML-007	**PROKKA_02512**	**Putative acyl-CoA thioester hydrolase**	15058	7.3	5
	PROKKA_56214	Peptidyl-prolyl cis-trans isomerase cyp18	21701	6.3	7
ML-008	PROKKA_92879	Major outer membrane protein P.IB precursor	39796	4.8	16
	PROKKA_12580	Peptidoglycan-associated lipoprotein precursor	18472	6.5	9
	**PROKKA_02512**	**putative acyl-CoA thioester hydrolase**	15058	7.3	4
	PROKKA_62359	bifunctional enoyl-CoA hydratase/phosphate acetyltransferase	15132	7.2	2
	PROKKA_134130	hypothetical protein	8378	8.3	2
	PROKKA_90774	Methionine synthase	23142	4.8	2

Table S1 continued:

ID	Database ID	Description	mW (Da)	pI (pH)	Peptides
ML-009	PROKKA_112699	Outer membrane protein W precursor	24708	5.9	6
	PROKKA_70441	hypothetical protein	13678	6.6	4
	PROKKA_77573	hypothetical protein	21589	10.5	3
	PROKKA_21264	Maf-like protein YceF	9493	4.7	2
	**PROKKA_130292**	**Esterase TesA precursor**	21383	5.6	5
	PROKKA_138839	ADP-ribose pyrophosphatase	23131	4.9	2
	PROKKA_90774	Methionine synthase	23142	4.8	4
ML-010	PROKKA_05026	hypothetical protein	8805	8.8	5
	**PROKKA_80567**	**Thioesterase superfamily protein**	12138	5.2	4
	**PROKKA_40999**	**Esterase TesA precursor**	21348	5.2	7
	PROKKA_90774	Methionine synthase	23142	4.8	2
	PROKKA_143609	Methyl-accepting chemotaxis protein McpS	58423	4.7	7
	PROKKA_138839	ADP-ribose pyrophosphatase	23131	4.9	2
	PROKKA_13373	Hypothetical protein	20831	9.7	7
ML-011	none				
ML-012	PROKKA_139397	Hypothetical protein	6677	5.5	1
	PROKKA_51656	Ribosomal protein S6 modification protein	6035	7.9	3
	PROKKA_96934	Peptidoglycan-associated lipoprotein precursor	19946	7.4	8
	PROKKA_137937	Hypothetical protein	23547	5.6	6

212 **Table S1 continued:**

ID	Database ID	Description	mW (Da)	pI (pH)	Peptides
ML-013	PROKKA_145948	Alkyl hydroperoxide reductase subunit C	13265	5.3	2
	PROKKA_13373	hypothetical protein	20831	9.7	6
	PROKKA_121960	Outer membrane protein P6 precursor	17810	5	7
	PROKKA_31930	Alkyl hydroperoxide reductase subunit C	20759	4.8	4
	PROKKA_05026	Hypothetical protein	8805	8.8	1
ML-014	PROKKA_121960	Outer membrane protein P6 precursor	17810	5	6
	PROKKA_39260	Outer membrane protein P6 precursor^APROKKA_138904 Outer membrane protein P6 precursor^APROKKA_152373 Outer membrane protein P6 precursor	17707	5	6
	PROKKA_13373	Hypothetical protein	20831	9.7	5
	PROKKA_52867	Hypothetical protein^APROKKA_80758 hypothetical protein	20674	9.7	4
	**PROKKA_12101**	**Putative acyl-CoA thioester hydrolase**	15226	6.4	3

213
